# Rapid Nanopore Whole-Genome Sequencing for Anthrax Emergency Preparedness

**DOI:** 10.3201/eid2602.191351

**Published:** 2020-02

**Authors:** Heather P. McLaughlin, Julia V. Bugrysheva, Andrew B. Conley, Christopher A. Gulvik, Blake Cherney, Cari B. Kolton, Chung K. Marston, Elke Saile, Erin Swaney, David Lonsway, Amy S. Gargis, Thiphasone Kongphet-Tran, Christine Lascols, Pierre Michel, Julie Villanueva, Alex R. Hoffmaster, Jay E. Gee, David Sue

**Affiliations:** Centers for Disease Control and Prevention, Atlanta, Georgia, USA (H.P. McLaughlin, J.V. Bugrysheva, C.A. Gulvik, B. Cherney, C.B. Kolton, C.K. Marston, E. Saile, D. Lonsway, A.S. Gargis, T. Kongphet-Tran, C. Lascols, P. Michel, J. Villanueva, A.R. Hoffmaster, J.E. Gee, D. Sue);; IHRC–Georgia Tech Applied Bioinformatics Laboratory, Atlanta (A.B. Conley);; Texas Department of State Health Services Laboratory, Austin, Texas, USA (E. Swaney)

**Keywords:** anthrax, Bacillus anthracis, bacteria, whole-genome sequencing, nanopore, emergency preparedness, bioterrorism and preparedness, zoonoses

## Abstract

Human anthrax cases necessitate rapid response. We completed *Bacillus anthracis* nanopore whole-genome sequencing in our high-containment laboratory from a human anthrax isolate hours after receipt. The de novo assembled genome showed no evidence of known antimicrobial resistance genes or introduced plasmid(s). Same-day genomic characterization enhances public health emergency response.

*Bacillus anthracis* causes anthrax, a deadly infectious disease, and is found worldwide, including areas of the United States. Naturally occurring anthrax outbreaks are reported annually in wild and domestic grazing animals, but human transmission is rare (*1*). Deliberate misuse of *B. anthracis* as a bioweapon could pose an immediate risk to human populations. In such instances, a timely response is critical to reduce morbidity and mortality rates.

After the anthrax incidents during 2001, the Centers for Disease Control and Prevention (CDC) published medical countermeasure recommendations for human anthrax treatment and postexposure prophylaxis using antimicrobial drugs, including amoxicillin, ciprofloxacin, doxycycline, levofloxacin, and penicillin (*2*). Most *B. anthracis* strains are susceptible to antimicrobial drugs; however, naturally occurring and engineered antimicrobial-resistant strains have been reported (*3**–**5*). Laboratory antimicrobial susceptibility testing (AST) by broth microdilution (BMD) remains the standard method to determine MIC values but requires >16 hours before results are available. During an anthrax emergency, rapid genomic characterization of the implicated *B. anthracis* strain(s) could identify sequences associated with drug resistance.

Single-nucleotide mutations in chromosomal *B. anthracis* quinolone resistance–determining regions of *gyrA*, *gyrB*, *parC*, and *parE* genes can lead to ciprofloxacin resistance, and gene acquisition can lead to tetracycline and doxycycline resistance (*3*,*4*,*6*). Penicillin resistance can result from a chromosomal mutation in the antisigma factor gene, *rsiP (**5*). Most *B. anthracis* strains carrying this signature *rsiP* mutation are resistant to penicillin and amoxicillin (*5*,*7*,*8*). Detection of known antimicrobial resistance (AMR) mutations or other novel gene insertions and deletions (indels) in the clonal *B. anthracis* genome signals genetic anomalies and could influence treatment and postexposure prophylaxis strategies.

Whole-genome sequencing (WGS) can identify gene indels, mutations, or previously undescribed genetic elements, including extrachromosomal plasmid DNA. However, common short-read sequencing (SRS) technologies have difficulty resolving bacterial genome structure because de novo assemblies yield multiple contigs. Long-read nanopore sequencing with the MinION device (Oxford Nanopore Technologies, https://nanoporetech.com) can resolve repetitive sequences and structural genomic rearrangements and enables complete bacterial genome finishing (*9*). Although MinION data are error-prone, especially in homopolymeric regions (*10*), compared with Illumina (https://www.illumina.com)–based SRS, it is available immediately during the sequencing run, enabling rapid assembly and analysis. The technology enables real-time sequencing, including direct pathogen identification from patient specimens, and holds the promise for future point-of-care applications that speed laboratory results reporting (*11*,*12*). Portable WGS instruments are advantageous for laboratories with limited space and remove the need to transfer DNA out of high-containment laboratories for sequencing, mitigating exposure risks to personnel (*13*).

CDC described a rapid nanopore sequencing approach and custom bioinformatics pipeline for *B. anthracis* that yielded complete chromosome and plasmid assemblies, and detected known AMR genes and mutations in avirulent laboratory strains (*13*). On the morning of August 2, 2019, our laboratory received a *B. anthracis* culture isolate (Ba0914) from a naturally occurring human anthrax case in Texas. Same-day laboratory WGS and bioinformatics analysis were performed. This study describes the laboratory work and demonstrates the usefulness of rapid WGS to inform time-sensitive public health responses.

## The Study

All laboratory work with the *B. anthracis* isolate and nanopore sequencing was performed inside a class II type A2 biological safety cabinet located in a US Federal Select Agent Program registered Biosafety Level 3 laboratory. We performed rapid nanopore sequencing as described by Gargis et al. (*13*), including silica membrane–based genomic DNA (gDNA) extraction, except that a bead-beating step was added to speed cell lysis. We extracted Ba0914 gDNA from colonies of an overnight agar culture in 75 min. Within the next hour, fluorometer and microvolume spectrophotometer measurements confirmed that the gDNA extraction was suitable for nanopore sequencing. We prepared a nanopore DNA sequencing library (Rapid Barcoding Sequencing Kit SQK-RBK004; Oxford Nanopore Technologies) and sequencing began <45 min later (MinKNOW, version 18.12.6; Oxford Nanopore Technologies).

Within ≈10 min of sequencing, nanopore data were ready for blastn analysis (https://blast.ncbi.nlm.nih.gov), which identified a 13.5-kb read with >91.5% sequence homology with *B. anthracis*. Approximately 120,000 live basecalled reads (average length 4,089 nt, average quality score/read 14.8) were generated in <5 hours of nanopore sequencing. We performed de novo genome assembly (Flye version 2.5; https://github.com) by using the first 120,000 nanopore reads and error-corrected with Medaka version 0.6.1 (https://pypi.org).

**Figure 1:**
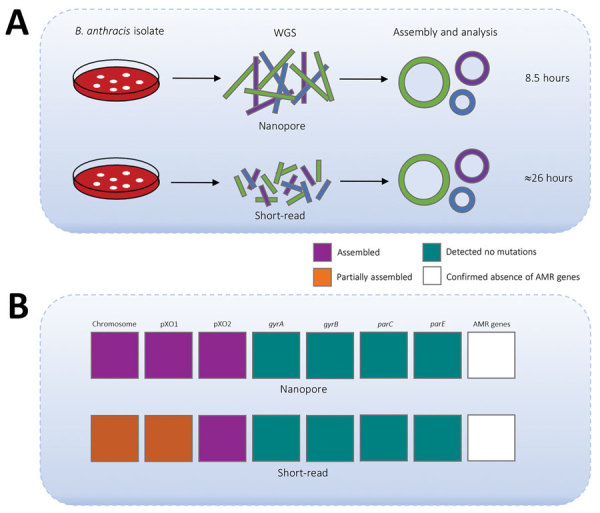
Time required to detect antimicrobial resistance markers in *Bacillus anthracis* strain Ba0914 by using WGS and summary of assembly results. A) Comparison of time to complete rapid nanopore (MinION) and short-read (iSeq) sequencing laboratory workflows. Workflows include DNA extraction, library preparation, WGS, and bioinformatics analysis. B) Comparison of nanopore-based and short-read sequencing–based data used to assemble the *B. anthracis* chromosome and plasmid sequences and to detect known AMR mutations and genes. Mutations associated with fluoroquinolone resistance in *B. anthracis* are located within the quinolone resistance–determining regions of *gyrA*, *gyrB*, *parC*, and *parE* genes. AMR genes contained in the Resfinder database (https://cge.cbs.dtu.dk) were queried against the assemblies. The *rsiP* mutation associated with penicillin resistance was not included. The nanopore assembly was generated by using the first 120,000 basecalled reads. AMR, antimicrobial resistance; WGS, whole-genome sequencing.

**Figure 2:**
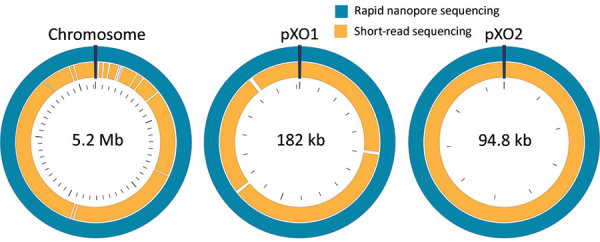
Circular maps of the whole-genome–sequenced *Bacillus anthracis* Ba0914 chromosome and 2 plasmids, pXO1 and pXO2, assembled by using rapid nanopore sequencing and short-read sequencing. (Maps are not to scale.)

The Ba0914 genome assembly contained single contigs for the chromosome and each plasmid, pXO1 and pXO2 (Figures 1, 2) with >54X average depth of coverage and shared >99.75% identity to the Ames Ancestor strain (GenBank accession no. AE017334) (Table). Thousands of indels and nearly 700 mismatches were detected (Table). Conventional BMD testing also began on August 2, 2019, according to guidelines of the Clinical and Laboratory Standards Institute (*14*), and susceptibility results were ready the following day.

**Table:** De novo whole-genome assembly metrics for sequencing of *Bacillus anthracis* strain Ba0914*

Aligned to	Mismatches	Indels	Contigs	Nucleotide identity, %; average-fold coverage		
				Chromosome	pXO1	pXO2
Ames reference strain						
Nanopore	677	6,411	3	99.83; 54	99.78; 192	99.80; 91
SRS	526	180	35	99.96; 115	99.94; 467	99.94; 220
SRS assembly						
Nanopore	166	6,305	NA	99.86	99.88	99.85

*Mismatches, indels, nucleotide identity, and average fold coverage for chromosomal and plasmid sequences of *B. anthracis* strain Ba0914 were determined on the basis of alignment with the Ames Ancestor reference strain assembly (top) or to the SRS-based Ba0914 strain assembly (bottom). The nanopore assembly was generated by using the first 120,000 live basecalled reads. Contigs, contiguous overlapping DNA segments; Indels, insertions and deletions; NA, not applicable; SRS, short-read sequencing.

We also performed Illumina-based SRS. We extracted DNA as described by Gargis et al. (*7*) and prepared the sequencing library (Nextera DNA Flex Library Kit; Illumina) for paired-end 2 × 150–bp sequencing by using the iSeq 100 (Illumina). We performed read filtering and assembly as described (*15*). The SRS-based assembly contained 32 more contigs than nanopore but with higher depths of coverage for the chromosome and plasmids (Table). Alignment to the Ames Ancestor reference strain yielded >99.9% genome identity, with fewer indels and mismatches. SRS-based approaches result in lower error rates and can more reliably detect single-nucleotide polymorphisms in *B. anthracis*, especially in homopolymeric regions, but a same-day laboratory workflow is currently not feasible (Figure 1, panel A). An alignment plot showed gaps in coverage of the chromosome (0.1%) and pXO1 (1.4%) caused by incomplete SRS-based assemblies (Figure 2). Alignment of the nanopore-based assembly to the SRS-based assembly resulted in >99.8% identity but with thousands of indels (Table). All sequencing data were submitted to GenBank under accession no. SAMN12588378.

Only 45 min of bioinformatics analysis (Pima version 01, https://github.com) using 120,000 basecalled nanopore reads was sufficient to assemble and confirm the absence of known AMR genes/markers associated with resistance to quinolones and tetracyclines (Figure 1, panel B) (*13*). We detected no mutations in *gyrA, gyrB*, *parC*, or *parE* genes, identified no AMR genes contained in the Resfinder database (https://cge.cbs.dtu.dk), and found no unexpected plasmids. SRS and bioinformatics analysis yielded analogous details about the AMR markers in Ba0914 (Figure 1, panel B). Only the SRS-based assembly, and not the nanopore assembly, was reliable for sequencing *rsiP*. Strain Ba0914 lacked mutations in the homopolymeric *rsiP* region that can confer penicillin resistance. Sequencing of regions containing repetitive nucleotide bases is a known limitation of nanopore technology and, consequently, detection of the *rsiP* mutation was excluded from AMR bioinformatics analysis.

Although genetic analysis is useful for detection of known AMR genes/markers in the *B. anthracis* genome, phenotypic susceptibility testing by BMD remains essential to detect functional resistance (*7*,*13*). By using the conventional BMD method, we found that strain Ba0914 was susceptible to ciprofloxacin, levofloxacin, tetracycline, doxycycline, penicillin, and amoxicillin.

## Conclusions

Real-time sequencing of the biothreat pathogen *B. anthracis* in a high-containment laboratory demonstrated the speed and usefulness of a rapid, portable nanopore sequencer during an emergency. Long-read sequencing could detect *B. anthracis*–specific DNA sequence from the culture isolate after only 3.5 hours. Although the nanopore-based assembly was error-prone when compared with the SRS-based assembly, as few as 8.5 hours would be required to find evidence of known AMR genes/markers or engineering, including gene insertions and extrachromosomal plasmids from *B. anthracis*. Although conventional AST remains essential for characterizing functional antimicrobial resistance in *B. anthracis*, nanopore sequencing provided same-day, on-site genomic characterization useful for an anthrax emergency response.
